# The Impact of pH on Fouling and Related Physicochemical Properties of Skim Milk Concentrate during Heat Treatment Using a Laboratory-Scale Fouling Rig

**DOI:** 10.3390/foods13193100

**Published:** 2024-09-28

**Authors:** Tara R. Murphy, Eoin W. Finnegan, Justyna Tarapata, Tom F. O’Callaghan, James A. O’Mahony

**Affiliations:** 1 School of Food and Nutritional Sciences, University College Cork, T12 Y337 Cork, Irelandtom_ocallaghan@ucc.ie (T.F.O.); 2 Dairy Processing Technology Centre, University College Cork, T12 Y337 Cork, Ireland

**Keywords:** milk concentration, fouling composition, viscosity, heat transfer

## Abstract

The objective of this study was to investigate the effect of pH (6.1, 6.3, 6.5, and 6.7) on heat-induced changes in concentrated skim milk as related to fouling in heat exchangers. Skim milk (30%, *w*/*w*, total solids) was recirculated in a laboratory-scale fouling rig at an initial target temperature of 85 °C for 90 min to simulate thermal processing and preheating of evaporated liquid concentrate feeds in dairy processing. This study investigated key changes in relevant physicochemical properties, such as viscosity, particle size, and sedimentation, as major contributors to fouling at lower pHs (6.1 and 6.3). Additionally, protein aggregation and calcium phosphate precipitation were identified as significant contributors to fouling deposits. Possible strategies to mitigate fouling were determined, including optimizing pH and adjusting heat treatment parameters to minimize protein denaturation and mineral deposition. The findings indicate that carefully controlling pH and processing parameters can greatly enhance the efficiency of milk concentration by evaporation and tailor finished product quality. Moreover, this study showed that monitoring of CIP solutions for protein content and turbidity provides valuable information on the intensity of fouling and the efficiency of cleaning.

## 1. Introduction

Skim milk powder is a key commodity in the dairy industry, widely produced and commercially significant. It is utilized as an ingredient in various food applications and is typically manufactured through the evaporation and spray drying of pasteurized skim milk [1,2]. In an effort to maximize efficiency and consistency of product quality, there has been a particular emphasis on optimization of unit operations, including evaporation, involved in the production of skim milk. The interplay between heat stability, viscosity, and fouling of skim milk concentrate is also directly linked with the techno-functional performance of the finished product powder (e.g., solubility and thermal stability). Evaporation involves heating the milk under vacuum to temperatures ranging from 55 to 70 °C. This heat treatment removes water from the milk and concentrates the liquid skim milk, typically achieving a total solids content of 48–50%, before it is spray-dried into a powder [3]. Evaporators are more energy-efficient than spray dryers, so the goal of evaporation is to concentrate the milk to high total solids before transferring it to the dryer. However, the viscosity of the concentrates often limits the amount of water that can be removed during evaporation [1]. The increase in concentrate viscosity during processing increases the chance of in-process fouling occurrence [4].

Fouling poses significant economic, operational, and environmental challenges for the dairy industry. The accumulation of deposits during production can increase thermal resistance by forming an insulating layer on the heat exchange surface, which thickens the barrier that heat must transfer through to reach the product, leading to a decline in equipment performance. This, in turn, obstructs fluid flow, increases pressure drop, and reduces the heat transfer coefficient [5,6]. As a result, the industry incurs higher operating costs due to more frequent cleaning requirements and increased energy consumption [7]. Fouling refers to the accumulation of undesired deposits in different parts of processing equipment, such as pipes, heat exchange surfaces, and other apparatus fittings [8], in addition to such foulant material also being present in the finished product. In dairy processing, the formation of fouling involves a complex process that encompasses the aggregation of proteins and the formation of insoluble calcium phosphate within the fluid. Protein aggregates primarily form deposits on heat transfer surfaces. In the study by Georgiadis and Macchietto [9], contributors to fouling were quantified, indicating that the deposition rate is influenced by the quantity of aggregated protein at the thermal boundary layer. Moreover, a thicker deposit corresponds to a greater temperature difference (∆T) between the heating medium and the product temperature. The authors [9] demonstrate that predictive models, under specific assumptions, can be developed to simulate fouling across various operating conditions. This capability is particularly useful for online monitoring, which aids in optimizing operating conditions and managing fouling more effectively. Therefore, further studies are needed to extend this understanding by examining factors affecting protein aggregation (such as milk pH) and thermal resistance in concentrated skim milk, which will help refine strategies for controlling fouling.

Another significant aspect of dairy fouling is the deposition of minerals, particularly calcium phosphate. As temperature increases, pH drops [10] and the solubility of calcium phosphate decreases, resulting in its precipitation and the formation of crystalline deposits on heat transfer surfaces [11]. Within fouling layers, calcium phosphate can bind to existing beta-lactoglobulin (β-lg) molecules, enhancing the stability of the fouling layer and facilitating protein–protein interactions through charge neutralization [12].

The cleaning protocols needed to remove fouling deposits are time-consuming, causing extended downtimes and significant environmental impact due to the use of chemicals, water, and heat [11,13]. Moreover, there is a risk of product quality deterioration, as the desired temperature may not be achieved [14], and the erosion of fouling layers can lead to finished product containing foulant with the potential to contribute to insolubility and sedimentation [15]. Biofilm formation also poses a threat to microbial quality, as bacteria, including spoilage and pathogenic microorganisms, can adhere to stainless-steel surfaces and reduce the microbial quality of the product [16].

Given the operational and environmental costs involved, it is important for the dairy industry to be able to measure or predict the presence, type, and intensity of fouling during thermal processes. This knowledge would assist in designing, improving, or adapting production processes to avoid unnecessary product or capital loss [17]. However, fouling is a complex process influenced by various parameters (pH, temperature, the presence of calcium ions and casein molecules, the surface characteristics of the equipment used, and the flow regime) that are challenging to measure accurately, making it difficult to quantify overall fouling resistance [18,19].

Hence, the objective of this research was to examine how pH affects the heat stability and heat-induced changes in concentrated skim milk, aiming to assess the suitability and interconnectedness of potential measures and indicators of fouling. This study categorizes fouling into two types: ‘adhered fouling’ and ‘non-adhered fouling’, with adhered fouling referring to a build-up of fouling deposits on thermal equipment and non-adhered fouling referring to any changes in product that are caused either by physicochemical reactions related to fouling or sedimentation of the fouling layer into the product.

## 2. Materials and Methods

### 2.1. Materials

Medium-heat skim milk powder (SMP) was provided by a local Irish dairy company (Arrabawn Co-op, Nenagh, Co., Tipperary, Ireland). The total protein, fat, ash, and carbohydrate of the SMP were 34.1, 0.80, 8.00, and 57.1% (*w*/*w*), respectively, as provided by the manufacturer. All chemicals and reagents, unless otherwise stated, were sourced from Sigma-Aldrich (Wicklow, Ireland) and were of analytical grade.

### 2.2. Reconstitution of Milk and pH Adjustment

Reconstituted skim milk was prepared by adding medium-heat SMP to ultrapure water over 1 h at 50 °C to attain either 9 or 30% (*w*/*w*) total solids (TS). The 9% concentration was chosen as a control to represent a typical total solids content in skim milk, while the 30% TS was selected to simulate more concentrated milk, reflecting conditions in the evaporator. Additionally, higher total solids concentrations are expected to increase milk viscosity, which raises the likelihood of fouling occurrence. Powder was added to water under constant stirring using an overhead stirrer (Eurostar 60 control, IKA, Staufen, Germany) with a spiral mixing element (Visco Jet 3x spiral mixing element d = 80 mm, Küssaberg, Germany). During rehydration, the stirring speed was gradually increased from 100 to 250 rpm. The solutions were stirred under the conditions described above for a further 3 h at 200 rpm, after which the solutions were cooled to 20 °C while magnetically stirring for pH adjustment. The pH of the skim milk solutions were measured using a pH meter (Mettler Toledo, FiveEasy pH/mV bench meter, F20, Greifensee, Switzerland) at 20 °C. The skim milk solutions (30% TS) that were later recirculated in the fouling rig were adjusted to pH 6.1, 6.3, 6.5, and 6.7 using 1 N NaOH and HCl as appropriate. Skim milk solutions (9 and 30% TS) were also adjusted to pH 6.0, 6.2, 6.4, and 6.6 for measuring heat coagulation time as a function of pH (described in Section 2.3). These solutions were magnetically stirred for a further 2 h, after which pH was readjusted to the target pH, if required, and followed by storage at 4 °C overnight with low-speed magnetic stirring to facilitate complete rehydration. After overnight rehydration, the samples were equilibrated to 22 °C for 2 h, after which the pH was checked and re-adjusted to the target pH, as required.

### 2.3. Heat Coagulation Time as a Function of pH

Heat coagulation time (HCT) of skim milk solutions at 9 and 30% (*w*/*w*) TS was determined at 120 °C as a function of pH in the range 6.0–6.7 using the method of Davies and White [20]. Samples (2 mL) were added to heat-resistant glass tubes and stoppered using a rubber bung, placed in a steel rack, and immersed in a silicone oil bath (Hettich ESP oil baths; Hettich Benelux BV, Geldermalsen, The Netherlands). The HCT was recorded as the time elapsed between immersing the sample in the oil bath and the visible detection of aggregates within the sample. Heat stability measurements were replicated six times for each pH level.

### 2.4. Fouling of Concentrated Skim Milk

**Figure 1:**
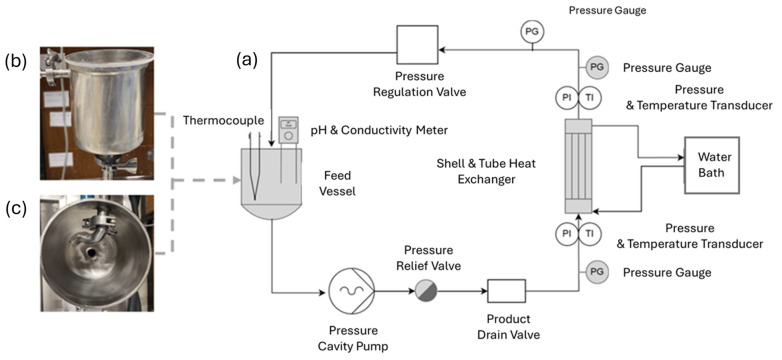
Process flow diagram of modified fouling rig set-up (**a**), the feed vessel from the modified fouling rig set-up (**b**), top-down view of the feed vessel from the modified fouling rig set-up, showing the feed tube designed to return product at an angle to promote turbulence without aeration or foaming (**c**). The shaded elements highlight the modifications compared to the set-up proposed by Hebishy et al. [21].

Fouling behavior of the skim milk solutions (4 L) at pH 6.1, 6.3, 6.5, and 6.7 was determined using a custom-built fouling rig, as per the method developed by Hebishy et al. [21], with some modifications (Figure 1a). In brief, the fouling rig used in this study consisted of a stainless-steel shell and tube heat exchanger with a 90 cm length and 5 cm internal diameter (Liam A. Barry Ltd., Cork, Ireland) and nine inner tubes with a diameter of 0.8 cm each. The heat exchanger was connected to a circulating water bath (Grant, LT ecocool™100, Cambridge, UK), which controlled the temperature of the water used as the heating medium. Two electronic pressure transducers (PR-33X, Keller-druck, Dorchester, UK), one located immediately before and one immediately after the heat exchanger, were used to measure temperature and pressure in line. Two analog pressure gauges, on either side of the electronic pressure transducers, were used as an additional measure of pressure. The unit had a stainless-steel feed vessel (4 L capacity; Liam A. Barry Ltd., Cork, Ireland) with a height of 17 cm and width of 15.5 cm (reducing down to 6 cm at the base of the vessel), rounded at the bottom (Figure 1b). The feed tube was redesigned to have a tangential entry port instead of directly down into the vessel as in the original design, with additional piping inside the vessel at an angle to promote turbulent flow within the vessel without causing aeration or foaming (Figure 1c). The feed vessel was connected directly to a positive displacement, progressive cavity pump (Torqueflow, Sydex, UK); the liquid flow rate through the heat exchanger was controlled by means of a variable speed drive. A three-way ball valve, located after the pump, allowed for the batch recirculation system to be drained at the end of the run. The pressure on the recirculating samples was set using a manual diaphragm flow control valve, which regulates milk flow by varying the valve opening to adjust the pressure, with pressure recorded using an analog pressure gauge (1107J486, WIKA, Los Angeles, CA, USA). The initial back pressure was set at 1 bar using the throttling valve and was not adjusted during the runs. pH and conductivity of the recirculating samples were measured using a pH/conductivity meter attached to a clear acrylic lid on top of the feed vessel (Metler Toledo, SevenGo Duo, SG23, Greifensee, Switzerland). The temperature of the samples was also measured using a thermocouple mounted to the feed vessel (Digitron 2024T Digital Thermometer Pt100, Port Talbot, UK). The fouling system was operated at a laminar flow rate (25 Hz) in batch recirculation mode. The samples were introduced to the fouling rig at an initial temperature of 25 °C, and the fouling experiments were conducted at 90 °C (temperature of the heating medium) to simulate thermal processing and preheating of spray dryer liquid concentrate feeds during SMP processing and to enable sufficient denaturation and deposit build-up on the heat exchanger [22,23]. The experiment was stopped after 90 min of recirculation, after which the recirculated samples were recovered for further analysis. After sample recovery, the fouling rig was visually inspected for fouling deposits, and any deposit formed was collected for further analysis.

The system was cleaned in place (CIP) using a standardized CIP protocol. The system was first rinsed using deionized water (4 L) for 10 min, after which a caustic wash was performed using 4 L of 1% (*v*/*v*) NaOH solution containing sodium hypochlorite (5%) (Ansep CIP, Ecolab, Co., Meath, Ireland) at 64 °C for 30 min to dissolve fat, protein, and carbohydrate deposits. After the caustic CIP step, the system was rinsed for 10 min with deionized water (4 L) to remove any residues of caustic. An acid wash was then performed using 4 L of 1% (*v*/*v*) acid solution containing nitric acid (>30%) and orthophosphoric acid (˂5%) (Horolith V, Ecolab, Co., Meath, Ireland) at 46 °C for 30 min to dissolve any remaining carbohydrate and mineral deposits. After the acid wash, the system was rinsed for 10 min with deionized water (4 L) to remove any residues of acid. The recirculated washes were recovered after each cleaning step for analysis of protein, ash, and turbidity.

### 2.5. Viscosity

The viscosity of the skim milk solutions (30%, *w*/*w*, TS; pH 6.1, 6.3, 6.5, 6.7) after each fouling experiment was measured using an AR-G2 controlled-stress rheometer (TA Instruments, Crawley, UK) equipped with a single wall concentric cylinder geometry (diameter 28.03 mm, length 42.96 mm). Samples (25 g), at 25 °C controlled by a Peltier apparatus (±0.1 °C), were subjected to a shear ramp from 0 to 300 s^−1^ for 4 min, followed by holding at 300 s^−1^ for 2 min, and a shear ramp from 300 to 0 s^−1^ for 4 min. Corresponding unheated samples of the skim milk solutions were used as controls.

### 2.6. Particle Size Distribution

Particle size distribution (PSD) of the skim milk solutions (30%, *w*/*w*, TS; pH 6.1, 6.3, 6.5, 6.7) after each fouling experiment (detailed in Section 2.4) was measured using a laser light diffraction unit (Mastersizer 3000 S, Malvern Instruments Ltd., Worcestershire, UK) equipped with a 300 reverse Fourier lens and He–Ne laser (λ of 633 nm). The samples were introduced to the dispersing unit using ultrapure water as dispersant to reach an obscuration of 10% (±1%). Analysis of PSD was performed using the generalized polydisperse model, with particle refractive index of 1.46, absorption of 0.1, and dispersant refractive index of 1.33. Non-heated samples of the skim milk solutions were used as controls.

### 2.7. Dry Sediment

Sediment was measured following a centrifugation method of Chen et al. [24], with minor modifications to adapt the method for high total solids material. After the fouling experiments, as detailed in Section 2.4, the skim milk solutions (30%, *w*/*w*, TS; pH 6.1, 6.3, 6.5, 6.7) were mixed to ensure a homogenous sample, and 20 g was accurately weighed and poured into a calibrated tube. The samples were then centrifuged (Sorvall RC5C Plus, Newtown, CT, USA) at 12,000× *g* for 1 h to remove protein aggregates. The high centrifugal force was necessary to sediment the particles of interest, and due to the increased viscosity of high-TS skim milk, the centrifugation time was extended to 1 h to ensure adequate separation. After the supernatant was removed, the sediment was oven-dried at 105 °C until a constant weight was obtained to determine its percentage dry weight. The moisture content (%, *w*/*w*) of the sediment was calculated by the difference in weight before and after oven drying the sediment. Unheated samples of the skim milk solutions were used as controls.

### 2.8. Composition of Skim Milk Solutions, Sediment, and Cleaning-in-Place Solutions

Samples of the skim milk solutions after the fouling experiments, the supernatant formed during the dry sediment method, and the CIP solutions used in cleaning cycles after the fouling experiments were analyzed for protein (%, *w*/*w*) and ash content (%, *w*/*w*). Protein was determined using the Kjeldahl method with a nitrogen-to-protein conversion factor of 6.38 (IDF, 2001). Ash content was determined by dry ashing in a muffle furnace (Nabertherm GmbH, Lilienthal, Germany) at 650 °C until a white ash was obtained (IDF, 2008).

The protein and ash content of the sediment was calculated as follows:

Skim milk solution crude protein − Supernatant crude protein = Sediment crude protein(1)

Skim milk solution ash − Supernatant ash = Sediment ash (2)

Samples of the skim milk solutions after the fouling experiments and the CIP solutions used in cleaning cycles after the fouling experiments were also analyzed for total solids (%, *w*/*w*) by oven drying at 104 °C until a constant weight was obtained (IDF, 2010).

### 2.9. Turbidity of Cleaning-in-Place Solutions

The turbidity of the CIP solutions was measured at 600 nm using a Cary 300 bio UV–visible spectrophotometer (Varian Inc., Palo Alto, CA, USA), which was equipped with a temperature control system. CIP solutions were measured for turbidity immediately after the cleaning cycles of the fouling rig.

### 2.10. Protein Profile Analysis

The protein profile of fouling material deposited in the heat exchanger by the skim milk solutions at pH 6.1 and 6.3 was analyzed using sodium dodecyl sulfate–polyacrylamide gel electrophoresis (SDS-PAGE), as described by Laemmli [25], with minor modifications. No fouling was observed in the heat exchanger after recirculating the skim milk solutions at pH 6.5 and 6.7; therefore, SDS-PAGE was not performed on these samples. Prepared samples were analyzed using pre-cast mini-protean Tetra Cell TGX 4–20% acrylamide gels (Bio-Rad Laboratories, Inc., Hercules, CA, USA) at a constant voltage of 180 V, under reducing and non-reducing conditions. The solid fouling material was dissolved in SDS buffer to obtain the desired protein load of 1 mg/mL in the individual gel loading wells. Five µL of SDS-PAGE broad range protein standard (Bio-Rad laboratories, Hercules, CA, USA) was loaded as a protein molecular weight standard. All gels were Coomassie-stained before being de-stained with a solution of water, methanol, and acetic acid, 50:40:10, respectively, until a bright background was reached. Stained SDS-PAGE gels were scanned using a desktop flatbed scanner (HP Scanjet G4010, HP, Leixlip, Ireland).

### 2.11. Statistical Analysis

All experimental analyses were conducted in triplicate, with samples produced from two independent trials for each pH, unless otherwise stated. Results are expressed as mean ± standard deviation. Analysis of variance (ANOVA; Tukey’s HSD test) was performed using R i386 version 4.0.2 (R Foundation for Statistical Computing, Vienna, Austria) to identify statistically significant differences between mean values for different samples. The level of significance was determined at *p* < 0.05 to determine whether statistically significant differences existed between the mean values.

## 3. Results and Discussion

### 3.1. Heat Stability of Skim Milk Solutions

**Figure 2:**
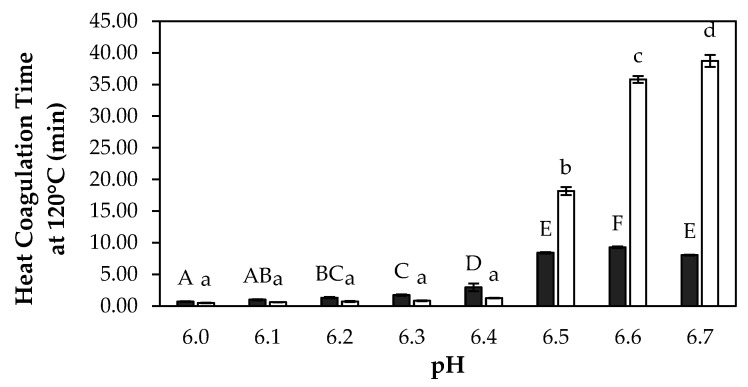
Heat coagulation time (HCT) at 120 °C as a function of pH for concentrated reconstituted skim milk at 30% total solids (TS) (■) and at 9% TS (□). Results are the means of data from three independent trials. ^A–F^ Mean values at 30% TS with different superscript letters are significantly different (*p* < 0.05). ^a–d^ Mean values at 9% TS with different superscript letters are significantly different (*p* < 0.05).

To determine the influence of solids concentration on the heat stability of the skim milk solutions, the heat coagulation time (HCT) was determined at 120 °C as a function of pH in the range 6.0–6.7, at both 9 and 30% (*w*/*w*) TS, with results presented in Figure 2. In the present study, at 9% (*w*/*w*) TS, the maximum HCT was observed at pH 6.7, and as the samples were acidified, the HCT decreased. The samples had significantly higher (*p* < 0.05) HCT at pH between 6.5 and 6.7, compared to pH 6.0–6.4. In addition, between pH 6.5–6.7, the HCT differs significantly (*p* < 0.05), varying from 18.2 to 38.7 min. For the skim milk solution at 30% (*w*/*w*) TS, the maximum HCT was observed at pH 6.6, and as the samples were acidified, the HCT decreased, but not as severely as the decrease in HCT observed on acidification of the skim milk at 9% (*w*/*w*) TS. The samples at 30% (*w*/*w*) TS had significantly higher (*p* < 0.05) HCTs between pH 6.5–6.7, compared to pH 6.0–6.4. In addition, between pH 6.5–6.7, the HCTs differ significantly (*p* < 0.05) in a small range, varying from 8.04 to 9.27 min, which was much lower than the HCTs observed at 9% (*w*/*w*) TS in the same pH range. The HCT at 30% (*w*/*w*) TS was slightly higher between pH 6.0 and 6.4 compared to 9% (*w*/*w*) TS, and the HCT of the 30% (*w*/*w*) TS samples at pH 6.3 and 6.4 were significantly higher (*p* < 0.05) than the samples at pH 6.0, 6.1, and 6.2, which was not observed at 9% (*w*/*w*) TS, where there were no significant differences (*p* > 0.05) in this pH range.

The pH range used was deemed essential for this study, as in-house preliminary trials demonstrated that the pH of skim milk decreased markedly as TS increased during evaporation, in agreement with findings of Anema [26], where a linear decrease in pH was observed when heating a 28.8% TS skim milk solution from 20 to 80 °C. As evaporation increases the dry matter content of milk, the decrease in pH can be attributed to the resultant increase in ionic components, precipitation of calcium phosphate, and resultant release of hydrogen ions from the casein micelle [27].

In general, the heat stability of milk follows two trends: a maximum HCT at the natural pH of milk (pH 6.6–6.8) and a local minimum at pH 6.9–7.0, or the heat stability profile shows an increase in HCT with increasing pH [28]. In this study, the control sample at 9% TS skim milk solution showed a maximum HCT at the natural pH of milk, which is the most dominant profile reported for skim milk [29]. The higher heat stability observed in the 9% TS samples between pH 6.5 and 6.7 is attributable to the stability of the casein micelles, as the pH is within the region of the native pH of milk, making them less prone to aggregation due to denatured whey proteins adsorbed to the micelle surface, forming a less calcium-sensitive steric barrier. The low heat stability of 9% TS samples between pH 6.0 and 6.4 can be attributed to ‘salt-induced’ coagulation. At low pH, the colloidal stability of casein micelles is lowered by a decrease in surface charge of casein micelles, reduced electrostatic repulsion, and a collapse of the hairy layer due to charge neutralization. This results in an increased tendency towards aggregation [30]. An increase in calcium activity and ionic strength by the dissolution of colloidal calcium phosphate (CCP) also contributes to an increase in calcium-mediated bridging between casein micelles [31]. The explanation for the differences in HCT between low pH (6.0–6.4) and high pH (6.5–6.7) samples at 30% (*w*/*w*) TS is essentially the same as that described above for the 9% (*w*/*w*) TS samples. Furthermore, Dumpler and Kulozik [32] reported that the HCT of skim milk decreased significantly (*p* < 0.05) with increasing TS content (from 10 to 35%), which aligns with our findings, showing significant differences in HCT at pH 6.5–6.7 between 9 and 30% TS skim milk. Moreover, efforts to improve the heat stability of concentrated skim milk by increasing pH through NaOH addition were shown to be less effective with increasing TS content [32]. Concentrated milk is generally recognized as being less heat-stable compared to unconcentrated milk. In another study, Dumpler et al. [33] developed a kinetic model for heat-induced coagulation of concentrated skim milk with TS content ranging from 12 to 33%, utilizing a Weibullian approach. In this model, heat coagulation is described as a two-step process: the destabilization of casein micelles followed by their aggregation, both of which are influenced by the milk’s environment and heating conditions. It has also been shown by Fourier transform infrared (FTIR) analysis that proteins in milk undergo considerable structural changes during concentration that make them more prone to destabilization reactions during heating [34]. Unlike unconcentrated milk, where protein polymerization plays a minor role, the higher concentration of whey protein in concentrated milk forms a complex network that leads to visible coagulation, making it a critical factor to consider [35]. Other factors that have been shown to lower the heat stability of concentrated skim milk include an increase in protein content [36,37]. All of these factors contribute to the increased susceptibility of concentrated skim milk to aggregation, therefore a lower heat stability than unconcentrated skim milk.

### 3.2. Fouling Rig

Skim milk solutions (30% TS) at pH 6.1, 6.3, 6.5, and 6.7 were chosen for fouling analysis by recirculation on the fouling rig based on the results from Section 3.1, as they represent the two distinct regions of the heat stability profile, i.e., samples at pH 6.1 and 6.3 displayed low heat stability, whereas samples at pH 6.5 and 6.7 displayed high heat stability.

#### 3.2.1. Inline pH and Conductivity Measurements

**Figure 3:**
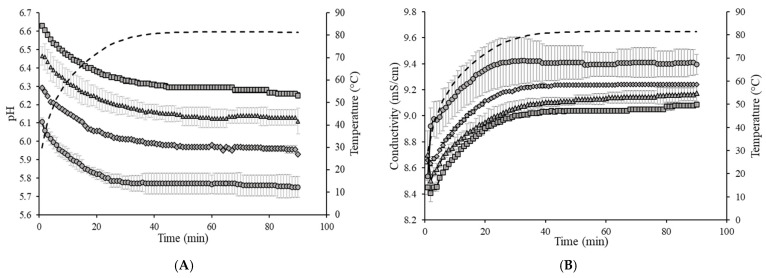
pH (**A**) and conductivity (**B**) as a function of time of concentrated skim milk solutions (30% *w*/*w*, total solids) at a starting pH of 6.1 (●), 6.3 (♦), 6.5 (▲), and 6.7 (■) during recirculation on the fouling rig at 85 °C. The temperature of the feed as a function of time (dashed line) is also shown. Results are the means of data from two independent trials. Error bars represent one standard deviation.

During recirculation of the skim milk samples on the fouling rig, pH and conductivity were measured at regular intervals to provide information on the effects of initial sample pH on the heat-induced changes during fouling (Figure 3A,B). Recirculation of the samples on the fouling rig resulted in a decrease in the pH of all samples (no significant differences (*p* > 0.05) in the extent of pH reduction between samples), with all samples decreasing in pH by ~0.36 units (Table 1). For all samples, the most extensive change in pH occurred during the initial heat-up phase of the fouling experiments (i.e., between 0 and 40 min), with a decrease in pH by ~0.31 units for all samples during this time. After the fouling rig experiments, the samples of concentrated skim milk were collected and cooled to 25 °C for measurement of pH; all samples did not return to their starting pH after fouling rig recirculation, with each sample reading ~0.1 units less than their initial pH.

A different trend was observed for conductivity, with recirculation of the samples on the fouling rig resulting in an increase in conductivity for all samples (Figure 3B). Before processing, all samples had approximately the same conductivity (~8.07 mS/cm), with no significant differences (*p* > 0.05) between them (Table 1). After recirculation in the fouling rig, the conductivity increased from 8.07 to 9.09 mS/cm for the sample at pH 6.7, which was significantly different (*p* < 0.05) from the conductivity increase for the samples at pH 6.3 and 6.5, increasing to 9.24 and 9.18 mS/cm, respectively. The sample at pH 6.1 had the largest increase in conductivity, being significantly different from all other samples (*p* < 0.05), increasing from 8.09 to 9.40 mS/cm during recirculation. In a similar trend to pH, the conductivity of all samples did not return to the starting value when cooled to 25 °C after the fouling rig trials, with all samples having a conductivity of ~8.23 mS/cm when cooled (no significant difference (*p* > 0.05) between samples, with an increase of ~0.16 mS/cm from the starting conductivity).

**Figure 4:**
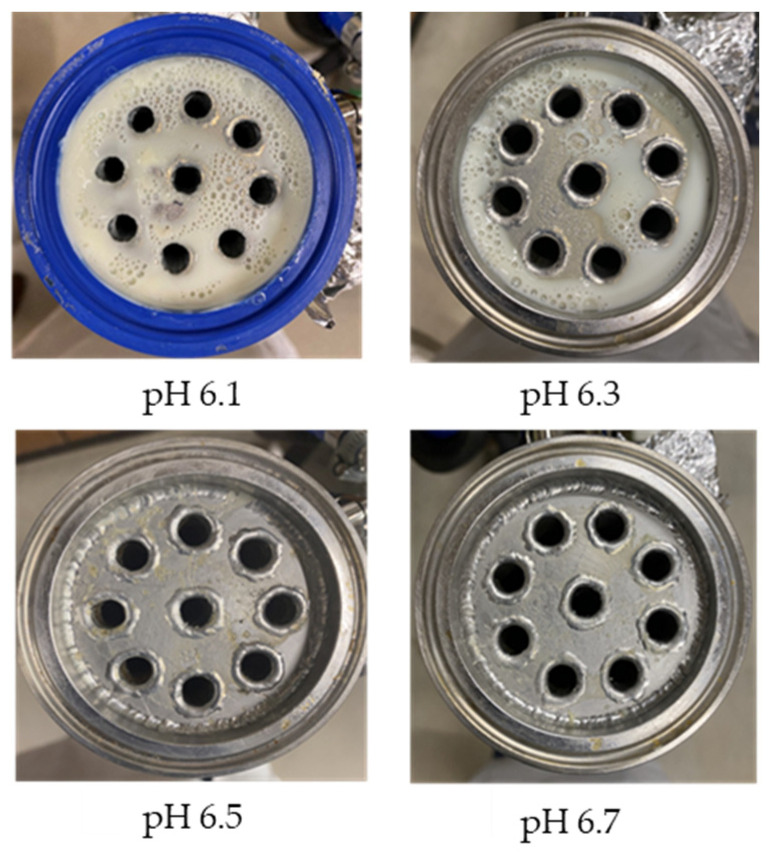
Images of the surface of the heat exchanger outlet after recirculation of concentrated skim milk solutions at 30% *w*/*w* total solids at pH 6.1, 6.3, 6.5, and 6.7 at 85 °C for 90 min.

The observed decrease in sample pH during recirculation can be attributed to the precipitation of CCP, since the solubility of calcium and phosphate ions decreases with increasing temperature [38] and the release of hydrogen ions that accompanies the precipitation of CCP causes the reduction in pH [39]. The samples at pH 6.1 and 6.3 displayed a build-up of adhered foulant (Figure 4), and it can be safely assumed that there was a higher amount of free calcium ions in the serum phase of these samples (compared to the samples at pH 6.5 and 6.7), as the pH of the samples before recirculation on the fouling rig was sufficiently low to allow solubilization of CCP and release of free calcium and phosphate ions into the serum phase [40]. Upon recirculation of the samples at pH 6.1 and 6.3 on the fouling rig, precipitation of CCP would still have occurred, causing a further decrease in pH, but the high amount of free calcium already in the serum phase would still be reactive and promote fouling through calcium-mediated bridging between casein micelles [41]. As the samples at pH 6.1 and 6.3 further decreased in pH upon recirculation on the fouling rig, the colloidal stability of casein micelles would have been lowered by a decrease in surface charge of casein micelles, a reduced electrostatic repulsion, and a collapse of the casein micelle surface hairy layer due to charge neutralization; combined, these effects result in an increased tendency towards aggregation [42]. Further aggregation of casein micelles caused by association of denatured whey proteins with κ-casein via intermolecular disulphide bonds and hydrophobic interactions contributes to the formation of an adhered fouling layer after recirculation of samples at pH 6.1 and 6.3 [43].

Milk has conductive properties due to its charged constituents, particularly mineral salts [44]. The electrical conductivity of milk is determined primarily by sodium and chloride ions [44] but also by other ions [45], with the distribution of salt fractions between soluble and colloidal forms being of particular importance to the electrical conductivity [46]. The observed increase in conductivity during recirculation of all samples on the fouling rig can be attributed to changes in equilibria of buffer systems and solubilization of casein-bound calcium and phosphorus salts in milk due to the acidification of milk on heating [47]. The greater increase (*p* < 0.05) in conductivity of the sample with an initial pH of 6.1 is due to the more extensive changes in mineral equilibrium and abundance of free ions as the pH of the sample decreased from 6.10 to 5.70 during recirculation on the fouling rig. These results show that conductivity is not directly related to fouling but rather is a co-product of changes in pH, which has been shown here to be directly related to the occurrence of fouling. Similar findings regarding the conductivity were described by Asselt et al. [6] in the study on monitoring of the CIP process with the focus on fouling. Therefore, inline measurements of pH (perhaps with conductivity measurements to coincide with pH readings) could be used in industry as an indicator of susceptibility to, and extent of, fouling during thermal processing.

#### 3.2.2. Temperature Difference

**Figure 5:**
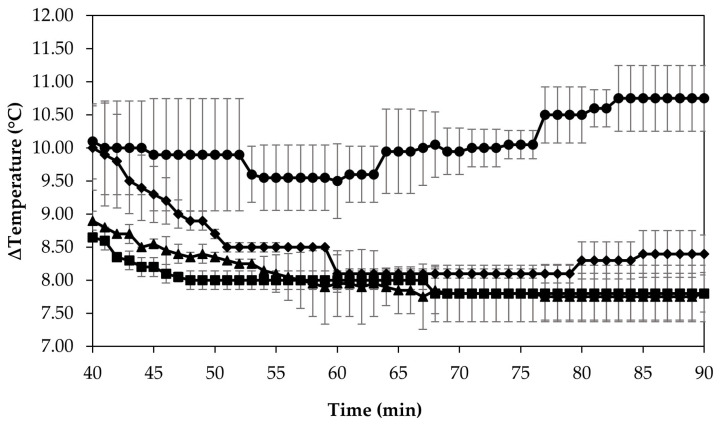
Difference in temperature of heating medium and product as a function of recirculation time for concentrated skim milk solutions at 30% *w*/*w*, total solids at pH 6.1 (●), 6.3 (♦), 6.5 (▲), and pH 6.7 (■), after the initial 40 min heat-up time of the fouling experiment. Results are the means of data from two independent trials.

During recirculation of the samples on the fouling rig, temperature was continuously measured to provide information on the efficiency of heat transfer across the stainless-steel heat exchange surface. The measured values for ∆T after the initial heat-up time (i.e., 40 min of recirculation) for each sample are shown in Figure 5. The skim milk samples at pH 6.5 and 6.7 displayed similar development in ∆T after 40 min of initial recirculation; there was a gradual decrease in ∆T before plateauing at ~68 min and reaching a final ∆T of 7.80 °C for both samples. The sample at pH 6.3 had a slightly different progression of ∆T compared to the samples at pH 6.5 and 6.7, whereby it decreased sharply before plateauing at 8.10 °C after 60 min, followed by a slight increase to 8.20 °C between 80 and 90 min. The sample at pH 6.1 had a very different progression of ∆T to all other samples; after a slight decrease in ∆T between 53 and 63 min, the ∆T increased steadily for the remainder of the experiment, peaking and plateauing after 83 min at 10.8 °C.

Upon visual inspection of the heat exchanger after the fouling experiments, no adhered foulant was observed after recirculation of the samples at pH 6.5 and 6.7 (Figure 4), demonstrating that the plateaued ∆T at 7.80 °C is indicative of no visual fouling for this system. An ‘acceptable’ ∆T, i.e., suggestive of no fouling, is process and product specific, and for the experimental configuration used in this study, it was determined through several preliminary trials that a ∆T between 2 and 8 °C is associated with no visible adhered fouling. The higher final ∆T at pH 6.3 can be attributed to the small amount of fouling deposit built up on the heat exchanger, impeding the heat transfer (Figure 4). The final ∆T after recirculation of the sample at pH 6.1 was significantly higher (*p* < 0.05) than the samples at a higher pH, and this coincides with the considerable amount of adhered foulant deposited on the heat exchanger by the sample at pH 6.1 (Figure 4 and Figure 5).

The ∆T provides information on the efficiency of heat transfer across the stainless-steel heat exchange surface. The thermal conductivity of the foulant is lower than that of the heat exchanger’s material, so its deposition results in the reduction in heat transfer rates [48]. Therefore, the temperature of the heating medium must increase in order to maintain product temperature for product quality and safety. This temperature increase can be used as an indirect measure of fouling. This concept was implemented and adapted in ultra-high temperature (UHT) fouling studies by Wadsworth and Bassette [49] and Hill et al. [50], where the temperature difference (∆T) between the heating medium and the treated milk leaving the system was monitored, with an increase in this ∆T parameter indicating fouling deposit build-up. The foulant material formed in this study at pH 6.1 and 6.3 was characteristic of ‘type-A deposit’ [51], which is a soft, curd-like material that forms at temperatures between 70 and 110 °C. A similar deposit was formed in a fouling study by Jeurnink et al. [52], and it was determined that the deposit consisted mainly of whey proteins, followed by minerals and caseins; the authors also reported that lowering the pH of milk results in severe fouling caused by casein micelles. Therefore, it can be hypothesized that the foulant formed in this study was attributable to mutual interactions of casein micelles due to aggregation facilitated by denatured whey proteins and calcium phosphate. This reaction, in conjunction with heat-induced disulphide bonding between whey proteins and κ-caseins, results in a significant degree of protein aggregation that can contribute to fouling [53]. No adhered foulant was formed in this study by the samples at pH 6.5 and 6.7, further supporting the hypothesis that the build-up of foulant is due to the association of denatured whey proteins and casein micelles. As the pH approaches the native pH of milk, the micelle surface charge will increase, reducing the tendency of the denatured whey proteins and casein micelles to associate [54].

### 3.3. Viscosity

**Figure 6:**
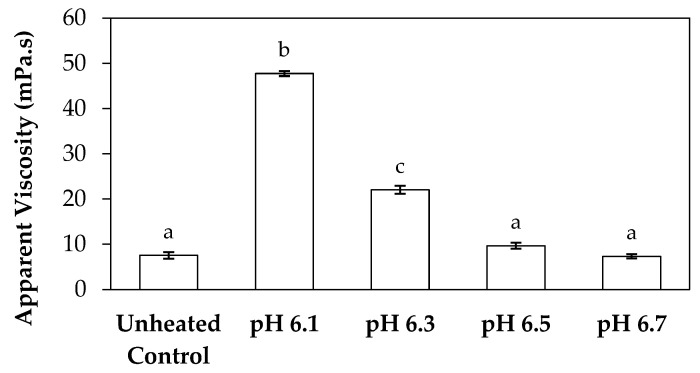
Apparent viscosity at a shear rate of 300 s^−1^ and 25 °C for concentrated skim milk solutions at 30% *w*/*w*, total solids, and pH 6.1, 6.3, 6.5, or 6.7 after 90 min of recirculation in the fouling rig at 85 °C. The unheated control was not subjected to heat treatment on the fouling rig. Results are the means of data from two independent trials. Mean values not sharing a common letter differ significantly (*p* < 0.05).

Measurements of viscosity of the concentrated skim milk samples at initial pHs of 6.1, 6.3, 6.5, and 6.7 were performed immediately after recirculation on the fouling rig (Figure 6). The viscosity of samples at pH 6.5 and 6.7 (9.68 and 7.34 mPa·s, respectively) after the fouling experiments did not differ significantly (*p* > 0.05) from that of the unheated control (7.54 mPa·s). Both the samples at pH 6.1 and 6.3 had significantly higher (*p* < 0.05) viscosity after the fouling experiments when compared to the unheated control, while also being significantly different (*p* < 0.05) from each other with viscosity values of 47.8 and 22.1 mPa·s for the sample at pH 6.1 and pH 6.3, respectively. More adhered foulant was visually apparent after recirculation of the skim milk sample at pH 6.1 when compared to the adhered foulant formed by the sample at pH 6.3 (Figure 4), which coincides with the higher viscosity of the sample at pH 6.1 after recirculation.

The significant increase in viscosity for the samples at pH 6.1 and 6.3 can be attributed to a greater extent of association of whey protein with the casein micelles during heat treatment, as reported by Anema et al. [30], who showed a similar effect when the pH of skim milk samples were adjusted to pH 6.5 and 7.1 prior to heat treatment, resulting in a higher viscosity being observed at pH 6.5 after heat treatment when compared to pH 7.1. A later study by Anema et al. [55] also reported the same phenomenon in a concentrated skim milk system, with a greater increase in viscosity being observed for samples heated at low pH. This more extensive increase in viscosity at low pH can be attributed to an increase in volume fraction and casein micelle voluminosity, as well as decreased electrostatic repulsion between micelles. When milk is heated, conformational changes in the tertiary structure of whey protein cause the proteins to denature and unfold, exposing the reactive thiol groups that interact with each other to form disulphide bonds, resulting in association with κ-casein, hydrophobic regions of the casein micelle, and other whey proteins. These interactions cause protein aggregation, which results in an increase in the volume fraction and voluminosity of the casein micelles [56,57].

Furthermore, concentrated dairy systems with high viscosity are associated with fouling of heat exchangers and reduction in heat transfer during evaporation [42,58]. As the samples at pH 6.1 and 6.3 resulted in adhered foulant in the heat exchanger (Figure 4) of the fouling rig, which did not occur for samples at pH 6.5 and 6.7, it can be hypothesized that the increased viscosity, driven by the denaturation/aggregation of proteins, is a key factor in the build-up of adhered foulant in thermal processing equipment.

It is also worth mentioning that offline viscosity measurements, like those conducted in this study, have several possible limitations, including the need for extended measurement times, which may lead to age gelation and associated errors. Additionally, offline measurements typically capture only a single point in the complex dynamic rheological profile of the sample [58]. In contrast, Process Analytical Technology (PAT) aims to overcome these limitations by enabling real-time monitoring of viscosity. Despite these challenges, the literature shows a significant positive correlation (r = 0.99, *p* < 0.05) between the dynamic viscosity measured by inline methods and the apparent viscosity obtained from offline methods in food processing [59].

### 3.4. Particle Size Distribution

**Figure 7:**
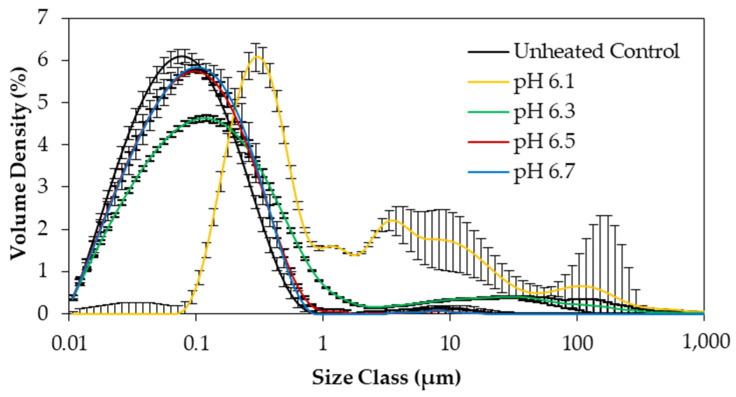
Particle size distribution of concentrated skim milk solutions at 30% (*w*/*w*) total solids after 90 min of recirculation in the fouling rig at 85 °C at pH 6.1, 6.3, 6.5, and 6.7 and unheated control.

Particle size analysis was conducted to assess the extent of protein aggregation in the concentrated skim milk solutions that fouled the heat exchanger of the fouling rig (pH 6.1 and 6.3), in comparison with samples that did not foul the heat exchanger (pH 6.5 and 6.7). The unheated control and the recirculated samples at pH 6.5 and 6.7 displayed monomodal particle size distributions (PSD’s), with one peak identified between 0.01 and 1 µm (Figure 7). At pH 6.3, an additional shoulder was observed between 1 and 300 µm, indicating a moderate level of protein denaturation and aggregation. The recirculated sample at pH 6.1 was polydisperse, displaying multiple large peaks between 0.1 and 1000 µm. The large standard deviations observed in the particle size distribution analysis of the recirculated sample at pH 6.1 can be attributed to the heterogeneous nature of the sample, with aggregates of varying size. There were no significant differences (*p* > 0.05) in volume mean diameter (D[3,4]) between the unheated control and the recirculated samples at pH 6.5 and 6.7 (Table 2). The recirculated samples at pH 6.1 and 6.3 showed significantly higher (*p* < 0.05) values for D[3,4], at 17.6 and 2.96 μm, respectively. The D[3,4] of the recirculated sample at pH 6.1 was significantly higher (*p* < 0.05) than that of the recirculated sample at pH 6.3 due to the dominance of very large, aggregated particles present (Table 2).

These results are in agreement with previous studies that showed an increase in particle size of heated skim milk with a decrease in pH [30,60]. It was also observed that the span for the recirculated sample at pH 6.1 was significantly larger (*p* < 0.05) than all other samples, as it displayed the most polydisperse PSD. It can be concluded there is a pH-dependent increase in particle size of concentrated skim milk on heating. At low pH (i.e., at pH 6.1 and 6.3), heat-induced whey protein association with casein micelles occurs, resulting in an increase in protein particle size due to aggregation of whey proteins with caseins, as well as with other whey proteins [54,61], which is reflected in the formation of adhered foulant in the heat exchanger of the fouling rig.

### 3.5. Composition of the Concentrated Skim Milk Solutions Following Fouling Rig Recirculation

To determine what components of the concentrated skim milk solutions contributed to fouling, measurement of the total solids, protein, and ash content of the samples before and after recirculation on the fouling rig was performed (Table 3). The total solids, protein, and ash content of the starting material was 30.3, 9.45, and 2.00%, respectively. The total solids content was significantly lower (*p* < 0.05) after recirculating the samples at pH 6.1, 6.3, 6.5, and 6.7, reducing to 23.3, 24.0, 24.6, and 24.9%, respectively. After recirculating the sample at pH 6.1, the protein content decreased significantly (*p* < 0.05) to 7.87%, whereas a less extensive, but significantly different (*p* < 0.05), change in protein content was observed after recirculating the sample at pH 6.3, with the protein content reducing to 8.02%. After recirculation at pH 6.5 and 6.7, the protein contents (8.20 and 8.50%, respectively) were significantly different (*p* < 0.05) from both the unheated control and the recirculated sample at pH 6.1, but not significantly different (*p* > 0.05) from the recirculated sample at pH 6.3. The ash content was also significantly lower (*p* < 0.05) after recirculating the samples at pH 6.1, 6.3, 6.5, and 6.7, reducing to 1.67, 1.74, 1.74, and 1.76%, respectively, with only the recirculated sample at pH 6.1 having a significantly different (*p* < 0.05) ash content from the other samples after recirculation. This indicates that minerals were a contributing factor to fouling caused by concentrated skim milk only at very low pH (pH 6.1), as both the mildly fouled (pH 6.3) and unfouled samples (pH 6.5 and 6.7) had similar ash contents after recirculation, further supporting that the fouling deposit was caused mainly by aggregated proteins, i.e., ‘type A fouling’. The reduction in ash content between the starting material and the samples drained from the fouling rig could be attributed to casein micelles in the fouling layer that contain a relatively large amount of minerals, as well as small amounts of solubilized calcium phosphate from the casein micelle facilitating protein–protein interactions [62,63].

**Figure 8:**
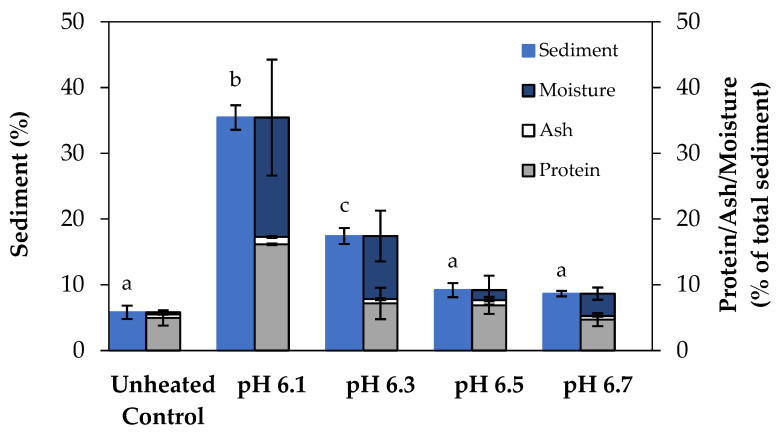
The relationship between pH and sediment formation following recirculation of concentrated skim milk solutions at 30% (*w*/*w*) total solids on the fouling rig at 85 °C for 90 min. The protein, ash, and moisture content of the sediments is expressed as a percentage of the total sediment formed for each sample. The unheated control was not subjected to any heat treatment. Results are the means of data from two independent trials. Mean values for sediment formation (%) with different superscript letters are significantly different (*p* < 0.05).

Furthermore, sediment was quantified in the current study as a measure of the extent of fouling. The sediment formation in the unheated control was significantly different (*p* < 0.05) from the heated samples with pH at 6.1 and 6.3, whereas samples at pH 6.5 and 6.7 did not differ significantly (*p* > 0.05) from the unheated control (Figure 8). The largest amount of sediment was formed at pH 6.1, followed by pH 6.3, with significantly different (*p* < 0.05) sediment values of 35.5 and 17.4%, respectively. The protein, ash, and moisture content of the sediment, presented as a percentage of the total sediment formed, provides insights into how the composition of the sediment formed in concentrated skim milk varies with pH (Figure 8). For all sediment formed, protein and moisture were found to be the main components. The unheated control had a very small percentage of moisture in the sediment, as the sediment formed was extremely compact and firm. As the pH of the concentrated milk was reduced, the amount of protein and moisture in the sediment increased. The sediment formed by the sample at pH 6.1 had the highest ash content; all other samples had ash contents comparable to the unheated control.

A transfer of the fouling deposit into evaporated milk products is a common occurrence in the dairy industry, with excessive sediment formation reducing product quality [64,65]. Sediment is formed during heating of concentrated skim milk by casein micelle and/or whey protein aggregation [66] and is an indicator of poor heat stability [24]. Previous studies have shown that the dry weight of sediment formed in samples of reconstituted milk heated and dialyzed at 115 °C for 30 min consisted of 50–60% protein, with all the major whey protein and casein fractions being identified in the sediment [67]. Lewis et al. [68] found that calcium ions are also a predominant factor influencing sediment formation, as ultraheat-treated milk with calcium chloride additions had significantly more sediment formation than milk without added calcium. The results of this study are aligned with those of On-Nom et al. [67], where no significant sediment was formed in heated samples of reconstituted skim milk if the pH was maintained above 6.3. The large amount of sediment formation in the low pH range can be attributed to protein aggregation, as evidenced in the earlier particle size analysis. Havea [69] used a similar method for determining sediment, in which milk protein concentrate was separated into two parts: the supernatant, which consisted of the soluble protein, and the sediment, which consisted of the insoluble protein, with the aggregated proteins defined as the insoluble protein, forming the sediment.

The higher percentage of protein present in the sediments formed at pH 6.1 and 6.3 can be attributed to extensive protein aggregation that occurs at low pH as the proteins become destabilized, consistent with previous hypotheses that sediment consists of mainly aggregated protein [66,67]. The high moisture content in the sediments formed in this study at pH 6.1 and 6.3 can be attributed to the presence of large protein aggregates, resulting in a less compact and porous sediment layer. These large aggregates could have prevented the sediment compacting as firmly as the unheated control during centrifugation, resulting in a large amount of trapped moisture in the sediment. The low pH of the sample would promote calcium activity by dissolution of the CCP [41], suggesting that the significant amount of sediment formed at pH 6.1 was due to calcium-mediated bridging between casein micelles as well as denatured whey proteins associated with κ-casein. Overall, these results coincide with previous results in studies of sediment formation in unconcentrated and concentrated milk on heating [66,67,68,69], which concluded that the amount of sediment formed in heated milk systems increases with decreasing product pH and that the sediment consists mainly of aggregated protein, followed by minerals (contributing to ash content). These studies, as well as the current study, have established a clear link between product instability and sediment formation. In this study, the samples that caused the most fouling also had the highest amount of sediment formation (i.e., samples at pH 6.1 and 6.3), showing that sediment formation is strongly linked with fouling.

### 3.6. Cleaning-in-Place Analysis

#### 3.6.1. Composition of the Recovered Cleaning-in-Place Solutions

**Figure 9:**
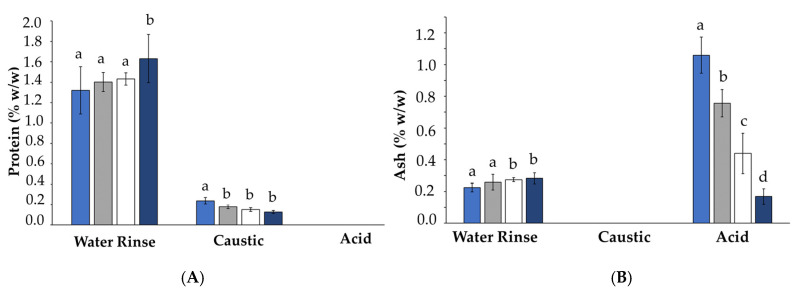
Protein content (**A**) and ash content (**B**) of cleaning solutions recovered after applying a cleaning-in-place protocol to the fouling rig after recirculation of concentrated skim milk solutions at 30% (*w*/*w*) total solids at 6.1 (■), 6.3 (■), 6.5 (□), and 6.7 (■) on the fouling rig at 85 °C for 2 h. Results are the means of data from two independent trials. Mean values within each individual cleaning step (i.e., water rinse) with different superscript letters are significantly different (*p* < 0.05).

The recovered cleaning-in-place (CIP) solutions obtained after applying the CIP protocols are illustrated in Table 4. CIP samples were analyzed for protein and ash content to further investigate the components of concentrated skim milk contributing to fouling (Figure 9). The protein and ash content of the water rinses differed significantly (*p* < 0.05) only after recirculation at pH 6.7, with the higher protein and ash content of the water rinse being attributed to any remaining deposit after recirculation being loosely bound [70].

A significant difference (*p* < 0.05) was observed in the protein content of the caustic wash after recirculation of the sample at pH 6.1 (0.24%), when compared to the protein content of the caustic washes after recirculation of samples at pH 6.3, 6.5, and 6.7. This demonstrates that considerably more protein adhered to the stainless-steel surfaces within the fouling rig at pH 6.1, further confirming that heat-induced protein denaturation contributes to fouling and that the reactions are promoted at low pH [71].

The ash content of the acid washes differed significantly (*p* < 0.05) between all samples, with the highest percentage ash being observed after the CIP regime for the recirculation of the skim milk solution at pH 6.1 (1.06%). A linear trend was observed for the ash content of the acid washes, whereby as the pH of the recirculated samples increased, the ash content of the acid washes decreased. This indicates that minerals play a more significant role in heat-induced reactions at low pH, further supporting the hypothesis that solubilized calcium phosphate is involved in the formation of fouling deposits caused by concentrated skim milk at low pH [72]. As the natural pH of milk (pH 6.7) decreases towards its isoelectric point, the net charge of the casein micelle decreases. This causes progressive protonation of organic and inorganic phosphate present in the casein micelles, resulting in dissolution of the CCP [73].

#### 3.6.2. Turbidity of the Recovered CIP Solutions

The results from turbidity analysis of recovered CIP solutions obtained after applying the CIP protocols (Table 5) were in good agreement with the protein and ash contents of the CIP solutions (Figure 9). The turbidity of the water rinses after recirculation of the samples at pH 6.5 and 6.7 (2.96 and 3.27, respectively) were significantly higher (*p* < 0.05) than the turbidity of the water rinses at pH 6.1 and 6.3 (2.91 and 2.91, respectively). This can be attributed to the higher protein and ash content of the water rinses at pH 6.5 and 6.7. The turbidity of the caustic wash used in the CIP regime after recirculation of the sample at pH 6.1 was significantly higher (*p* < 0.05) than all other samples, which can be attributed to the significantly higher (*p* < 0.05) amount of protein dissolved by the caustic wash after recirculation of concentrated skim milk at pH 6.1. The turbidity of the acid washes was significantly different (*p* < 0.05) for all samples, following a linear trend with a decrease in turbidity being observed with increasing sample pH, which is in agreement with the trend observed in ash content of the acid washes. These results show a direct correlation between the turbidity and protein and ash content of the CIP solutions, i.e., the more fouling material dissolved by the CIP solutions, the more turbid the solutions were. Other fouling studies have monitored the cleaning procedure after fouling has occurred through turbidity measurements, proving it to be a convenient and efficient method for promptly assessing the cleaning effectiveness. Guerrero-Navarro et al. [74] found that analyzing turbidity to compare the effectiveness of different cleaning strategies for fouling served as an efficient and effective indicator of the cleaning process’s advancement, facilitating optimization efforts. Van Asselt et al. [6] employed real-time turbidity monitoring via spectrophotometry in an automated CIP system to evaluate the elimination of protein fouling. Similarly, Fickak et al. [75] utilized turbidity and conductivity measurements during the rinsing phase to assess the effectiveness of the cleaning process. The results of this study, coinciding with other fouling research, show that measuring turbidity of CIP solutions after cleaning cycles can be used to analyze the cleaning effectiveness after fouling, i.e., critical turbidity values could indicate the degree of protein or mineral deposit removal. It is important to ensure cleaning regimes are effective in completely removing the fouling deposits, as any remaining deposit in the system will facilitate the formation of biofilms and allow for fouling reactions within the product to occur more quickly, as the proteins and minerals have a reactive surface to bind to [76].

### 3.7. Protein Profile of Foulant Material

**Figure 10:**
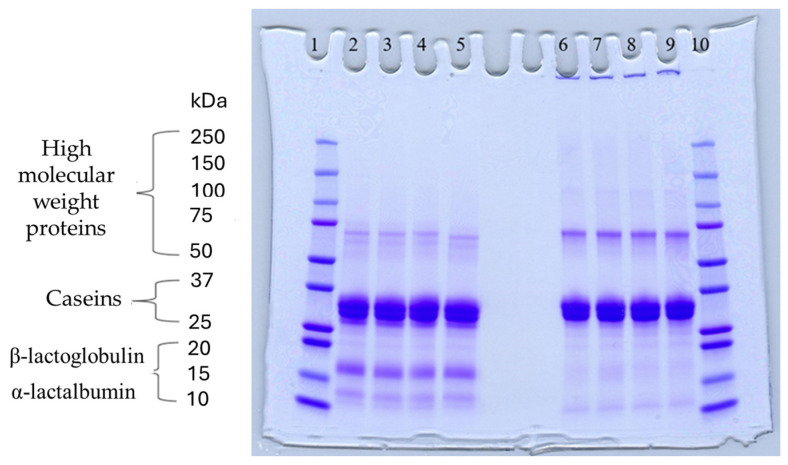
Sodium dodecyl sulfate–polyacrylamide gel electrophoresis (SDS-PAGE) electrophoretograms of fouling material recovered from the heat exchanger of the fouling rig after recirculation of concentrated skim milk solutions at 30% *w*/*w* total solids at pH 6.1 and 6.3 at 85 °C for 90 min: lanes 1 and 10, molecular weight marker; lanes 2–3 and 6–7, foulant at pH 6.1; lanes 4–5 and 8–9, foulant at pH 6.3. Lines 1–5: reducing conditions; lines 6–10: non-reducing conditions.

The fouling material built up in the heat exchanger after recirculation of the concentrated skim milk at pH 6.1 and 6.3 (Figure 4) was analyzed electrophoretically under reducing and non-reducing conditions (Figure 10). From SDS-PAGE analysis, it can be observed that the protein profile of the fouling material deposited by the samples at pH 6.1 and 6.3 was similar. The bands corresponding to the caseins were the strongest in intensity in both the reducing and non-reducing gel, indicating that casein is one of the most dominant proteins in the fouling deposit formed by concentrated skim milk. The intensity of bands corresponding to β-lg was markedly more prominent in the reducing gel compared to the non-reducing gel, indicating that denatured β-lg was a key component of the large aggregates present in the loading well of the non-reducing gel. The bands corresponding to α-lactalbumin (α-lac) were slightly more intense in the reducing gel, indicating that a small extent of denatured α-lac was also present in the fouling material. During the fouling process for concentrated milk, β-lg undergoes unfolding and denaturation due to heating, exposing the reactive thiol group (-SH) present in β-lg, which can interact with other milk proteins such as casein and α-lac, leading to the formation of aggregates and deposits [77,78,79,80]. Comparing the bands between 50 and 75 kDa in the reducing and non-reducing gels indicates the presence of protein aggregates in this region. In the non-reducing gel, there is only one major protein band below the 75 kDa band; however, in the reducing gel, this band is less intense and there are also additional bands, signifying the presence of other minor whey proteins such as bovine serum albumin (BSA) and lactoferrin; these bands were essentially absent in the non-reducing gel, indicating that these proteins could also have reacted in forming the large aggregates present in the loading well of the non-reducing gel. BSA, like β-lg, possesses a single free -SH group, which can participate in intramolecular disulfide bonds with other protein molecules [81], triggering the formation of large aggregates. The differences in the intensity of the bands between the reducing and non-reducing gels indicate that there was a significant amount of disulfide bonding between the caseins and denatured whey proteins. It was reported that whey proteins account for more than 50% of the fouling deposits in type A fouling, with β-lg being the dominant protein due to its sensitivity to heat [82,83].

## 4. Conclusions

This study illustrated the intricate relationships among pH, heat stability, viscosity, particle size, sedimentation, and fouling in concentrated skim milk during thermal processing. As the initial pH of the concentrated skim milk solutions decreased, the heat stability decreased. Under the conditions of this study, fouling was induced in concentrated skim milk samples at pH 6.1 and 6.3 (i.e., those with low heat stability), but not at pH 6.5 and 6.7. Severe fouling was visibly evident and confirmed by a high ∆T for the sample at pH 6.1 and less so for the sample at pH 6.3. Although no significant differences in the degree of pH and conductivity change during fouling rig recirculation were observed between samples, it is important to note that all samples showed reductions in pH of ~0.36 units, highlighting the importance of monitoring pH during thermal processing. The concentrated skim milk samples that displayed fouling had higher viscosity, larger particle size distribution, and more sediment formation when compared to the unheated control and the samples that did not demonstrate fouling. Analysis of the composition of the concentrated skim milk solutions after recirculation on the fouling rig revealed reduced protein and ash content compared to the initial material, with this reduction being more significant at pH 6.1 and 6.3. These findings, along with compositional analysis of cleaning-in-place (CIP) solutions and SDS-PAGE analysis of the adhered foulant, demonstrated that the fouling deposits consisted primarily of aggregated protein, with the relatively high ash content of the CIP solutions indicating some calcium phosphate binding facilitated by the low pH. Through a combination of analytical approaches and tools, a deeper understanding of fouling was achieved, aiding in the identification of strategies to mitigate its occurrence in the future. One such strategy could include the addition of calcium-binding salts such as citrates and phosphates, which can help maintain milk stability and reduce fouling. Moreover, this study established the efficacy of the custom-fabricated fouling rig as a powerful tool for investigating the fouling behavior of concentrated skim milk and cleaning of dairy heat exchangers. The rig’s modular design facilitated the generation of deposits under high-temperature, short-time conditions for compositional characterization and assessment of cleaning-in-place solutions. In the future, the fouling rig could be employed to explore potential process modifications aimed at reducing the occurrence of fouling.

## Figures and Tables

**Table 1:** Inline measurements of pH and conductivity during recirculation of concentrated skim milk solutions at 30% (*w*/*w*) total solids at a starting pH of 6.1, 6.3, 6.5, and 6.7. Measured changes (∆) in these parameters are shown. The abbreviations ‘t_0_’ and ‘t_90_’ indicate the measurements at the start (0 min) and end (90 min) of the fouling experiment.

	pH					Conductivity (mS/cm)					
t_0_	t_90_	Cooled to 25 °C after Run	∆ during Run	∆ 0–40 Min	∆ 40–90 Min	t_0_	t_90_	Cooled to 25 °C after Run	∆ during Run	∆ 0–40 min	∆ 40–90 min
6.1	5.70 ^a^ ± 0.13	6.00 ^a^ ± 0.00	0.36 ^a^ ± 0.08	0.34 ^a^ ± 0.08	0.02 ^a^ ± 0.00	8.09 ^a^ ± 0.07	9.40 ^a^ ± 0.08	8.21 ^a^ ± 0.01	1.31 ^a^ ± 0.01	0.87 ^a^ ± 0.69	0.04 ^a^ ± 0.01
6.3	5.93 ^b^ ± 0.00	6.20 ^b^ ± 0.00	0.36 ^a^ ± 0.01	0.30 ^a^ ± 0.01	0.06 ^a^ ± 0.00	8.09 ^a^ ± 0.00	9.24 ^b^ ± 0.00	8.25 ^a^ ± 0.00	1.15 ^b^ ± 0.00	0.57 ^a^ ± 0.03	0.01 ^a^ ± 0.00
6.5	6.11 ^c^ ± 0.07	6.36 ^b^ ± 0.09	0.36 ^a^ ± 0.02	0.31 ^a^ ± 0.01	0.05 ^a^ ± 0.03	8.04 ^a^ ± 0.02	9.18 ^b^ ± 0.06	8.24 ^a^ ± 0.04	1.14 ^b^ ± 0.08	0.41 ^a^ ± 0.22	0.07 ^a^ ± 0.03
6.7	6.25 ^d^ ± 0.01	6.58 ^c^ ± 0.04	0.38 ^a^ ± 0.01	0.32 ^a^ ± 0.00	0.06 ^a^ ± 0.01	8.07 ^a^ ± 0.05	9.09 ^c^ ± 0.02	8.23 ^a^ ± 0.02	1.02 ^c^ ± 0.07	0.58 ^a^ ± 0.07	0.06 ^a^ ± 0.01

Results are the means of data from two independent trials. Mean values within a column not sharing a common superscript letter differ significantly (*p* < 0.05).

**Table 2:** Particle size distribution parameters for concentrated skim milk solutions at 30% *w*/*w* total solids after 90 min of recirculation in the fouling rig at 85 °C. The unheated control was not subjected to heat treatment on the fouling rig.

Sample	D[2,3]	D[3,4]	Dv(10)	Dv(50)	Dv(90)	Span
	(µm)					
Unheated Control	0.05 ± 0.00 ^a^	0.24 ± 0.09 ^a^	0.02 ± 0.00 ^a^	0.08 ± 0.00 ^a^	0.26 ± 0.02 ^a^	3.06 ± 0.18 ^a^
pH 6.1	0.46 ± 0.01 ^b^	17.6 ± 7.68 ^b^	0.19 ± 0.00 ^b^	0.65 ± 0.05 ^b^	25.2 ± 5.88 ^b^	38.5 ± 6.41 ^b^
pH 6.3	0.07 ± 0.01 ^a^	2.96 ± 0.62 ^c^	0.03 ± 0.00 ^a^	0.13 ± 0.00 ^a^	0.94 ± 0.13 ^a^	7.26 ± 0.94 ^c^
pH 6.5	0.06 ± 0.00 ^a^	0.19 ± 0.06 ^a^	0.03 ± 0.00 ^a^	0.09 ± 0.00 ^a^	0.31 ± 0.01 ^a^	3.19 ± 0.11 ^a^
pH 6.7	0.06 ± 0.00 ^a^	0.18 ± 0.03 ^a^	0.02 ± 0.00 ^a^	0.09 ± 0.00 ^a^	0.29 ± 0.01 ^a^	3.16 ± 0.07 ^a^

Results are the means of data from two independent trials. Mean values within a column not sharing a common superscript letter differ significantly (*p* < 0.05). D[3,4] = volume-weighted mean particle diameter; D[2,3] = surface-weighted mean particle diameter; Dv(10) = particle size below which 10% of sample volume is found; Dv(50) = particle size below which 50% of sample volume is found; Dv(90) = particle size below which 90% of sample volume is found.

**Table 3:** Protein, ash, and total solids content (% *w*/*w*) of concentrated skim milk solutions at 30% *w*/*w* total solids at pHs 6.1, 6.3, 6.5, and 6.7 after heating through recirculation through the fouling rig at 85 °C for 90 min. Unheated control was not subjected to any heat treatment. Samples analyzed after heating are abbreviated to ‘AH’.

Sample	Total Solids	Protein	Ash
		(%*w*/*w*)	
Unheated Control	30.3 ± 0.17 ^a^	9.45 ± 0.34 ^a^	2.00 ± 0.02 ^a^
pH 6.1 AH	23.3 ± 0.48 ^b^	7.87 ± 0.77 ^b^	1.67 ± 0.07 ^b^
pH 6.3 AH	24.0 ± 0.82 ^bc^	8.02 ± 0.72 ^c^	1.74 ± 0.02 ^c^
pH 6.5 AH	24.6 ± 0.82 ^bc^	8.20 ± 1.29 ^c^	1.74 ± 0.04 ^c^
pH 6.7 AH	24.9 ± 0.32 ^c^	8.50 ± 0.46 ^c^	1.76 ± 0.07 ^c^

Results are the means of data from two independent trials. Mean values with different superscript letters within a column are significantly different (*p* < 0.05).

**Table 4:** Images of cleaning solutions recovered after applying a cleaning-in-place (CIP) protocol to the fouling rig after recirculation of concentrated skim milk solutions at 30% *w*/*w*, total solids at pH 6.1, 6.3, 6.5, and 6.7 on the fouling rig at 85 °C for 2 h.

CIP Solution	pH of Concentrated Skim Milk Sample Recirculated before CIP Regime			
	pH 6.1	pH 6.3	pH 6.5	pH 6.7
Water Rinse	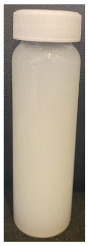	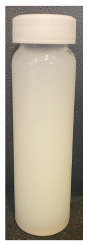	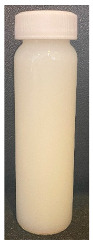	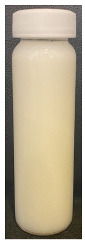
Caustic Wash	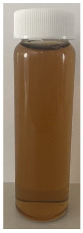	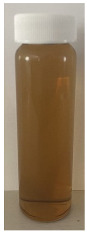	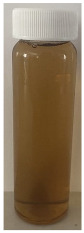	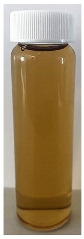
Acid Wash	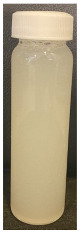	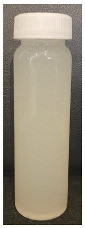	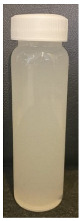	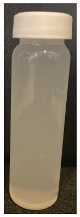

**Table 5:** Turbidity (optical density at 600 nm) of cleaning solutions recovered after applying a cleaning-in-place protocol to the fouling rig after recirculation of concentrated skim milk solutions at 30% *w*/*w* total solids at pH 6.1, 6.3, 6.5, and 6.7 on the fouling rig at 85 °C for 2 h.

	Turbidity (OD at 600 nm)		
Circulated Milk pH	Water Rinse	Caustic Wash	Acid Wash
pH 6.1	2.91 ± 0.02 ^a^	0.14 ± 0.01 ^a^	0.76 ± 0.13 ^a^
pH 6.3	2.91 ± 0.00 ^a^	0.10 ± 0.02 ^b^	0.63 ± 0.18 ^b^
pH 6.5	2.96 ± 0.01 ^b^	0.09 ± 0.01 ^b^	0.31 ± 0.02 ^c^
pH 6.7	3.27 ± 0.01 ^c^	0.09 ± 0.01 ^b^	0.18 ± 0.03 ^d^

Results are the means of data from two independent trials. Mean values within a column with different superscript letters are significantly different (*p* < 0.05).

## Data Availability

The original contributions presented in the study are included in the article, further inquiries can be directed to the corresponding author.
